# Editorial: Haptic training simulation, volume III

**DOI:** 10.3389/frobt.2025.1550392

**Published:** 2025-01-27

**Authors:** Carlos Rossa, Xiaojun Chen, Troy McDaniel, Arnaud Leleve

**Affiliations:** ^1^ Department of Systems and Computer Engineering, Faculty of Engineering and Design, Carleton University, Ottawa, ON, Canada; ^2^ Institute of Biomedical Manufacturing and Life Quality Engineering, School of Mechanical Engineering, Shanghai Jiao Tong University, Shanghai, China; ^3^ School of Manufacturing Systems and Networks, Arizona State University, Mesa, United States; ^4^ University Lyon, INSA Lyon, Université Claude Bernard Lyon 1, Ecole Centrale de Lyon, CNRS, Ampère, UMR5005, Villeurbanne, France

**Keywords:** haptics, hands-on training, tactile, simulation, kinesthetic

This third edition of the Haptic Training Simulation Research Topic presents papers highlighting future research in haptic-enabled technologies for surgical training. These works demonstrate the importance of such technologies fostering motor and sensory skills while reducing cognitive load, keeping costs low, and streamlining design. See Lelevé et al. (2020) and Chen et al. 2022 editorials on the Research Topic Haptic Training Simulation.

Low Cost and Fast Design. Research on haptic training simulation often proposes advanced simulators for specific medical gestures without effective off-the-shelf solutions. This leads to complex, costly solutions, limiting affordability for large audiences. They therefore target experienced trainees needing haptic training for rare situations or surgeons rehearsing complex operations. However, real-world evidence shows a need for simple, affordable solutions for classrooms at all educational levels. These require fast design and low technical requirements to reduce costs and ease large-scale deployment. This edition presents papers exploring this approach. González-Mena and Neri et al. illustrate this need, using a common simulation framework combining a design process and software development kit for haptic training simulation. This framework, VIS-HAPT, stems from previous research and helps design “visuo-haptic” experiments using a computer and generic haptic device. Both papers showcase applications for virtual physics experiments in undergraduate and engineering classrooms, providing statistical studies on acceptance and utility. Neri et al. further test training effectiveness, showing enhanced learning through simulation. They also highlight important design criteria like simplicity, readability, and interactivity. These simulators aim to provide engaging tools for learning Physics more dynamically than traditional lectures.

Dual Users. Another way to make use of haptic devices for enhancing hands-on training is illustrated in Zhang et al. In this study, the haptic devices are not used to interact with the objects in a virtual world as in González-Mena and Neri et al., but to reproduce the expert gestures on the hands of the trainees. More precisely, the surgical tools handled by the expert are connected to individual haptic devices, each one recording in real time its connected tool trajectory. These trajectories are sent to the trainees’ devices, which, in turn, guide the tools of the trainees. Thus, trainees can follow in their hands the expert tool trajectories, instead of only watching them and reproducing them on their own as usual. This experimental study suggests that haptic feedback superimposed on the trainee’s motions can facilitate the performance of novice operators experiencing moments of difficulty, which was something that was already observed in other works (see the discussion of Zhang et al. for references). Even if the small sample size and use of a simple task limit the generalizability of their findings, this study illustrates that haptic training can be realized without any 3D virtual world, which requires accurate modeling for realistic haptic rendering of a complex task.

Tactile Feedback. In Ratschat et al., the authors designed a shape exploration experiment to evaluate the effectiveness of multimodal tactile and kinesthetic feedback on shape perception. Sixteen participants were involved to reproduce different two-dimensional shapes with diverse characteristics in free space after exploring the shapes with two haptic feedback conditions: 1) kinesthetic feedback only and 2) kinesthetic plus tactile feedback. The kinesthetic feedback mechanism was implemented through an adapted single-degree-of-freedom SenseGlove Nova mechanism with an integrated electromagnetic brake. And tactile feedback was provided with a cable-driven platform mounted on the fingertip. To measure the participants’ ability to perceive and reproduce the rendered shapes, the authors recorded the time participants spent exploring and reproducing the shapes and the error between the rendered and reproduced shapes after exploration and assessed the workload and motivation with questionnaires. Experimental results show that in a virtual shape exploration task without visual feedback, providing tactile and kinesthetic feedback is associated with more accurate and careful shape reproduction compared to exploring shapes with only kinesthetic feedback. Besides, the addition of tactile feedback does not seem to reduce the time spent during exploration, nor does it have an effect on motivation or workload. Thus, combining haptic and kinesthetic feedback could create more realistic virtual environments that may lead to better training results and easier transfer to real-world tasks, having implications across a variety of applications and training scenarios.

Vibrotactile. Similarly, in Boutin et al., the authors combined haptic (vibrotactile) feedback through the use of a haptic glove with a VR simulator for mixed-reality surgical training. They specifically focused on the potential for enhanced sensory feedback within VR. The authors chose to investigate External Ventricular Drain Placement (EVD), a common neurosurgery procedure, as a starting point. Experimental results demonstrated the simulator’s accuracy, even though one major limitation was a lack of kinesthetic feedback. Like Ratschat et al., this work shows the potential to create more realistic mixed-reality environments that could extend beyond surgical applications.

Kinesthetic. Force feedback plays a vital role in developing surgical skills, yet many virtual reality simulators lack this feature, creating a significant disparity between physical trainers and their digital counterparts, potentially limiting their effectiveness. In Abinaya and Manivannan, the authors take a different approach and use haptic feedback as an assessment metric for surgical training, focussing on laparoscopic surgery using a virtual reality simulator. By incorporating haptic feedback, they replicate the forces between the tool and the tissue, which directly correlate to tissue trauma. A virtual laparoscopic force model is incorporated into the simulator and used to determine the just noticeable differences of the laparoscopic grasping force. The results suggest that a simple linear model is sufficient for gripper force feedback, and a non-linear model does not affect the force perception. Expert laparoscopic surgeons agree that haptic feedback improves learning performance, and the force model improves the accuracy of object interaction during the gripping task.

Innovative approaches to enhance surgical training must foster motor and sensory skills while reducing cognitive burden, lowering costs, and being conducive to faster design processes. This Research Topic, the third on the Research Topic of Haptic Training Simulation, underscores the potential of haptic-enabled virtual reality tools in shaping the future of surgical education and improving patient outcomes.
